# N-glycopeptide Signatures of IgA_2_ in Serum from Patients with Hepatitis B Virus-related Liver Diseases*[Fn FN2]

**DOI:** 10.1074/mcp.RA119.001722

**Published:** 2019-09-09

**Authors:** Shu Zhang, Xinyi Cao, Chao Liu, Wei Li, Wenfeng Zeng, Baiwen Li, Hao Chi, Mingqi Liu, Xue Qin, Lingyi Tang, Guoquan Yan, Zefan Ge, Yinkun Liu, Qiang Gao, Haojie Lu

**Affiliations:** ‡ Liver Cancer Institute, Zhongshan Hospital, and Key Laboratory of Carcinogenesis and Cancer Invasion (Ministry of Education), Fudan University, Shanghai 200032, China; § Institutes of Biomedical Sciences, Fudan University, Shanghai 200032, China; ¶ Beijing Advanced Innovation Center for Precision Medicine, Beihang University, Beijing 100083, China; ‖ Key Lab of Intelligent Information Processing of Chinese Academy of Sciences (CAS), Institute of Computing Technology, CAS, Beijing 100190, China; ** Department of Gastroenterology, Shanghai General Hospital, Shanghai Jiaotong University, Shanghai 201620, China; ‡‡ Department of Clinical Laboratory, First Affiliated Hospital of Guangxi Medical University, Nanning 530021, Guangxi, China; §§ School of Biomedical Informatics, The University of Texas Health Science Center at Houston, Houston, TX 77030; ¶¶ State Key Laboratory for Novel Software Technology, Nanjing University, Nanjing 210046, China; ‖‖ Department of Chemistry, Fudan University, Shanghai 200433, China; *** NHC Key Laboratory of Glycoconjugates Research, Fudan University, Shanghai 200032, China

**Keywords:** Glycoproteomics, Glycomics, Liver Disease, Biomarker: Diagnostic, Mass Spectrometry, 40-kDa Band, Glycopeptide, HBV-Related Liver Diseases, IgA2, Mass Spectrometry

## Abstract

N-glycosylation alteration has been reported in liver diseases. Characterizing N-glycopeptides that correspond to N-glycan structure with specific site information enables better understanding of the molecular pathogenesis of liver damage and cancer. Here, unbiased quantification of N-glycopeptides of a cluster of serum glycoproteins with 40–55 kDa molecular weight (40-kDa band) was investigated in hepatitis B virus (HBV)-related liver diseases. We used an N-glycopeptide method based on ^18^O/^16^O C-terminal labeling to obtain 82 comparisons of serum from patients with HBV-related hepatocellular carcinoma (HCC) and liver cirrhosis (LC). Then, multiple reaction monitoring (MRM) was performed to quantify N-glycopeptide relative to the protein content, especially in the healthy donor-HBV-LC-HCC cascade. TPLTAN^205^ITK (H5N5S1F1) and (H5N4S2F1) corresponding to the glycopeptides of IgA_2_ were significantly elevated in serum from patients with HBV infection and even higher in HBV-related LC patients, as compared with healthy donor. In contrast, the two glycopeptides of IgA_2_ fell back down in HBV-related HCC patients. In addition, the variation in the abundance of two glycopeptides was not caused by its protein concentration. The altered N-glycopeptides might be part of a unique glycan signature indicating an IgA-mediated mechanism and providing potential diagnostic clues in HBV-related liver diseases.

## Abstract (precis)

^18^O/^16^O labeling N-glycopeptide quantification and MRM have been performed to investigate aberrant N-glycopeptides in HBV-related liver diseases. TPLTAN^205^ITK (H5N5S1F1) and (H5N4S2F1) of IgA_2_ were significantly elevated in serum from patients with HBV infection and even higher in LC, as compared with healthy donor. In contrast, the two glycopeptides of IgA_2_ fell back down in HBV-related HCC. The altered N-glycopeptides might be part of a unique glycan signature indicating IgA-mediated mechanism.

## Graphical Abstract

**Figure:**
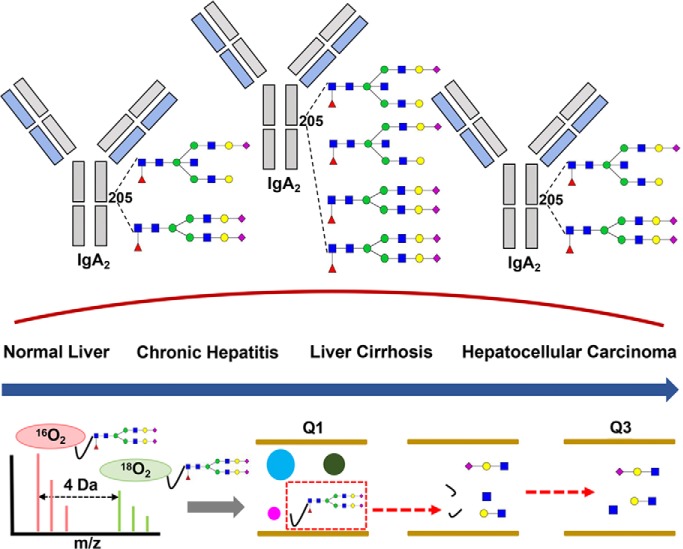

**Highlights**

- ^18^O/^16^O labeling N-glycopeptide quantification for 40-kDa band in HCC and LC.
- MRM verification of aberrant N-glycopeptides in healthy-HBV-LC-HCC cascade.
- TPLTAN^205^ITK (H5N5S1F1) and (H5N4S2F1) of IgA_2_ change in liver diseases.
- Variation in two N-glycopeptides abundance not caused by protein concentration.

N-glycosylation is the complex posttranslational modification displayed on many proteins and plays important roles in physiopathological processes. It has been reported that serum glycoproteins are mainly produced by the liver (1), while immunoglobulins are produced by the immune system, for example, IgG, IgM, IgA, IgE, and IgD are secreted by B cells during an immune response (2). Aberrant N-glycosylation is implicated in the development and progression of cancer, such as cell signaling and communication, tumor cell dissociation and invasion, cell-matrix interactions, and immune modulation (3, 4). Unique alterations in tumor-associated N-glycosylation can provide distinct biomarkers, and there are several intrinsic advantages due to rapid responses to diseases and significant and amplified changes (5–8). For example, a “glycomics” biomarker based on profiling of the N-glycans from the total serum protein can be used to assess the risk of hepatocellular carcinoma (HCC)^1^ development in compensated cirrhosis (9). *Wisteria floribunda* agglutinin^+^-Mac 2-binding protein showed diagnostic ability to detect cirrhosis of the native liver (10). Enhanced fucosylation of acute-phase proteins such as haptoglobin (11, 12) have been reported in HCC. A recent review by Zhu *et al.* summarized glycoproteomics markers of HCC especially based on mass spectrometry (MS) approaches (13).

HCC often develops from hepatitis B virus (HBV) infection and cirrhotic livers in China, and liver cirrhosis (LC) is the strongest predisposing factor (14). It was reported that core-fucosylation was important for HBV infection of hepatoma cells through HBV-receptor-mediated endocytosis (15), and specific HBsAg major hydrophilic region N-glycosylation mutations were implicated in HBV immune escape in a high endemic area (16). Characterizing the heterogeneity of glycans in HBV-related liver diseases would lead to a better understanding of the molecular pathogenesis of liver damage and cancer, providing novel diagnostic, prognostic, and therapeutic clues.

Based on MS, intact glycopeptide analysis that includes both glycan structure and glycosylation site information can distinguish glycosylation patterns on individual proteins (17). Recently, novel MS platforms, such as IsoTaG (18), NGAG (19), SugarQb (20), and pGlyco (21), facilitate comprehensive and integrated characterization of glycopeptides for further understanding of their biological role (22). For example, quantitative analysis revealed higher amounts of O-GlcNAc glycosylation on transcription factors c-JUN (c-JUN is a member of the Jun family and is a component of the transcription factor AP-1) and JUNB (JUNB is a basic region-leucine zipper transcription factor belonging to the Jun family), which were also up-regulated at the protein level, in activated T cells (23).

Labeling and label-free methods are available for MS-based quantification of biological samples. For labeling methodologies, the quantitative results can be obtained simultaneously by comparing the abundance of the isotopologues, including enzyme labeling (for example, trypsin catalyzed ^18^O labeling), chemical labeling (for example, iTRAQ), and metabolic labeling (for example, SILAC (stable isotope labeling with amino acids in cell culture)). Among them, enzymatic ^18^O labeling only require in the presence of ^18^O-water, without extra reagents, additional steps, side reactions, and chromatographic isotope effects (24, 25).

Serious challenges remain for N-glycopeptide analyses in diseases, such as complexity and diversity of N-glycans (26), and lack of validation. It was reported the majority of plasma glycoproteins were 24 glycoproteins, over half of them with the molecular weights of 40–55 kDa (40-kDa band) (27). In this study, a cluster of serum glycoproteins in 40-kDa band were chosen to assess their intact N-glycopeptides and evaluate its potential for noninvasive monitoring of HBV-related liver diseases. Compared with the whole serum, analyses of target group decrease the complexity of biological samples and increase accuracy of quantification; compared with a single molecule, analyses of a target group enable simultaneous measurements of related molecules using fewer samples and shorter period. In addition, combination of an ^18^O/^16^O labeling N-glycopeptide method and multiple reaction monitoring (MRM) was performed to confirm glycopeptide alterations, which can improve the quantitative power and increase the understanding of their functional impact of the observed changes.

## EXPERIMENTAL PROCEDURES

### 

#### 

##### Experimental Design and Statistical Rationale

First, an N-glycopeptide method based on ^18^O/^16^O C-terminal labeling was used to obtain comparisons of serum from patients with HBV-related HCC and LC: (1) with 45 biological repeats, N-glycopeptides that occurred at least 10 times (QC1), and passed stringent filtering criteria (QC2, FDR<1%; QC3, 0<score interference ≤ 0.3 and 0.8<similarity ≤ 1) were considered; (2) another 37 biological repeats were performed to confirm N-glycopeptides alterations. Thus, in total, there were 82 biological comparisons based on ^18^O/^16^O C-terminal labeling; each comparison contained one HCC serum (pooled from 10 randomly selected HCC individuals) and one LC serum (pooled from 10 randomly selected LC individuals).

Then, Tier 3 of MRM analyses was applied in this study, where glycopeptide abundance was divided by unique peptide abundance to separate out the contribution of protein concentration: (1) For MRM verification of LC and HCC patients, crude serum was obtained from 10 HCC individuals and 10 LC individuals; purified IgA was also obtained from these samples; and (2) for MRM measurement of healthy donor-HBV-LC-HCC cascade, crude serum was obtained from another 10 independent HCC individuals, 10 independent LC patients, 10 individuals with HBV infection, and 10 normal subjects; purified IgA was also obtained from these samples for measurement of healthy donor-HBV-LC-HCC cascade.

##### Patient Samples

The serum specimens were all obtained from The First Affiliated Hospital of Guangxi Medical University, including 100 HBV-related LC, 100 HBV-related HCC, 10 HBV patients, and 10 healthy donors. All blood samples were handled identically: 5 ml of venous blood were drawn from each individual from each group (drawn before any treatments and surgery), placed in room temperature for 1 h until coagulated, and serum was recovered by centrifugation at 3000 rpm for 10 min and stored in aliquots at −80 °C until used. This study was approved by the Research Ethics Committee of The First Affiliated Hospital of Guangxi Medical University. Informed consent was obtained from all patients and normal controls. The clinical data of the patients are provided in Supplemental Table S1. Patients with autoimmune diseases or other virus infection were excluded in this study.

##### Protein Digestion and Glycopeptide Enrichment

Two hundred μg of standard glycoprotein haptoglobin (Calbiochem, San Diego, CA) and 10 μl serum were separated by 10% SDS-PAGE and the protein bands were visualized with Coomassie blue. Then, the 40–55 kDa band (from the lower limit of 55 kDa (marker) to lower limit of 40 kDa (marker)) was excised, cut into small pieces, and destained with buffer (50% acetonitrile (ACN):100 mm NH_4_HCO_3_ = 1:1, v/v). These gel pieces were reduced with 5 mm Tris (2-carboxyethyl) phosphine hydrochloride in 100 mm NH_4_HCO_3_ for 30 min at 37 °C and alkylated with 55 mm iodoacetamide in 100 mm NH_4_HCO_3_ for 30 min at room temperature in the dark. Sequencing-grade modified trypsin (Promega, Madison, WI) was added at an enzyme to a substrate ratio of 1:50 (w/w) overnight at 37 °C. The tryptic peptides were extracted with a solution of ACN, H_2_O and trifluoroacetic acid (50%, 49.9%, and 0.1%, respectively) and lyophilized. The tryptic peptides were applied to Glycopeptide Enrichment Kit (Novagen, Darmstadt, Germany) according to manufacturer's protocol.

##### ^16^O/^18^O Incorporation into the C Termini of Peptides

Immobilized trypsin (Thermo Scientific, Rockford, IL) was added into each tube at an enzyme-to-substrate ratio of 1:5 (v/w) and dried in a vacuum centrifuge. The two lyophilized aliquots were redissolved with 10 μl ACN (20% v/v) compounded in H_2_^16^O/H_2_^18^O (97%, Cambridge Isotope Laboratories, Andover, MA) 100 mm ammonium acetate buffer, respectively, to catalyze the labeling of tryptic peptides C-terminally at 37 °C for 24 h. One μl formic acid (FA) was added for complete quenching, and immobilized trypsin beads were removed by centrifuge columns (Pierce, Rockford, IL).

##### Nano-Liquid Chromatography Tandem MS

The experiment was performed on an EASY-nano-LC 1000 system (Thermo Scientific) connected to an Orbitrap Fusion mass spectrometer (Thermo Scientific) equipped with an online nano-electrospray ion source. Five μl ^18^O-labeled and 5 μl ^16^O-labeled digest were combined, and 4 μl of the mixture were loaded onto the trap column (PepMap C18, 100 μm × 2 cm), with 15 μl solvent A (solvent A: water with 0.1% FA; solvent B: ACN with 0.1% FA) and subsequently separated on the analytical column (PepMap C18, 75 μm × 25 cm) with a linear gradient, from 1% B to 25% B in 60 min and from 25% B to 45% B in 20 min. The column was re-equilibrated at initial conditions for 10 min. The column flow rate was maintained at 300 nl/min. The electrospray voltage of 2.0 kV *versus* the inlet of the mass spectrometer was used. The parameters for Orbitrap Fusion mass spectrometer were: (1) MS: scan range (*m/z*) = 350–2000 Da; resolution = 120,000; AGC (automatic gain control) target = 500,000; maximum injection time = 50 ms; included charge state = 2–6; dynamic exclusion duration = 15 s; (2) higher energy collisional dissociation -MS/MS: isolation window = 4 *m/z*; detector type = Orbitrap; resolution = 15,000; AGC target = 400,000; maximum injection time = 200 ms; normalized collision energy = 30%; stepped collision mode on, energy difference of ± 10%.

##### Intact Glycopeptide Identification by pGlyco and Quantification by pQuant

For glycopeptide identification, each raw MS/MS datum was converted to “mgf” format and searched by pGlyco 2.0 (Version 2017.09.25) (http://pfind.ict.ac.cn/software/pGlyco/index.html) for ^16^O- and ^18^O-labeling, respectively. Parameters for database search of intact glycopeptide are as follows: both mass tolerance for precursors and fragment ions were set as ± 20 ppm. The protein databases were from Swiss-Prot reviewed, date March 2015, with species of *Homo sapiens* (20,215 entries). The enzyme was full trypsin. Maximal missed cleavage was 2. Fixed modification was carbamidomethylation on all Cys residues (C +57.022 Da). Variable modifications contained oxidation on Met (M +15.995 Da). The N-glycosylation sequon (N-X-S/T, X ≠ P) was modified by changing “N” to “J” (the two shared the same mass). The glycan database was extracted from GlycomeDB (www.glycome-db.org). For ^18^O-tag searching, additional peptide modification (any C-term +4.008 Da) was added. All identified spectra could be automatically annotated and displayed by the software tool gLabel embedded in pGlyco, which facilitates manual verification. In addition, pGlyco supplied glycopeptide FDR estimation. Glycopeptide FDR estimation was used for quality control, and the N-glycopeptides below the criterion of a 1% glycopeptide FDR were considered to be identified in this study. Then, ^18^O/^16^O-labeled glycopeptides were quantified by pQuant (http://pfind.ict.ac.cn/software/pQuant/index.html). It calculates ^18^O/^16^O glycopeptides ratio based on a pair of least interfered isotopic chromatograms. The workflow of pQuant consists of three steps: extraction of glycopeptide signals, quantitation calculations of glycopeptides, and stringent quality control based on score interference (0<score interference ≤ 0.3) and similarity (0.8<similarity ≤ 1). Score interference represents the interference level of coeluting ions of similar *m/z* values in MS; similarity represents similarity between the experimental isotopic distribution and a theoretical isotopic distribution in MS.

##### Purification of IgA and MALDI-TOF/TOF MS

The column (Thermo Scientific) was packed with 400 μl CaptureSelect IgA affinity resin (Thermo Scientific) and equilibrated with 5 ml wash buffer (PBS, pH 7.4). Then, 200 μl human serum were loaded onto the column. After 5-ml wash buffer, 5-ml elution buffer (0.1 m glycine, pH 3.0) was used to collect fraction. Elution fractions were neutralized with 500 μl 1 m Tris, pH 8.0, ultrafiltrated (Merck Darmstadt, Germany) and separated by 10% SDS-PAGE. The band of IgA was excised, reduced, alkylated, and trypsin treated. The tryptic peptides were extracted and applied to 5800 MALDI-TOF/TOF MS (AB SCIEX, Framingham, MA). One μl (∼1 μg/μl) peptides solution was combined with 1 μl (4 μg/μl) matrix CHCA (α-cyano-4-hydroxycinnamic acid) (Sigma-Aldrich, Schnellendorf, Germany), and submitted for acquisition of MALDI spectra in the positive mode. The datasets obtained were converted into mgf files using Msconvert of the ProteoWizard software (v3.0.10273). Peptide and protein identification were performed by Mascot (Version 2.3.0, Matrix Science) based on Database (SwissProt 57.15 (515,203 sequence entries; 181,334,896 residues)). The search included oxidation (M) as a variable modification and carbamidomethyl (C) as a fixed modification. The precision tolerance was ± 0.2 Da for peptide masses and ± 0.2 Da for fragment ion masses. Trypsin was chosen as enzyme, and the number of missed cleavage sites was assigned to be 1.

##### MRM Analyses

MRM analysis of individual sample was performed on a 6500 QTRAP mass spectrometer (AB SCIEX) coupled with an Eksigent 425 (AB SCIEX) nano HPLC. Two target glycopeptides and one unique peptide of IgA_2_ were chosen and optimum transitions for each glycopeptide/peptide were determined (Supplemental Table S2). Standard curves were prepared with natural human IgA_2_ protein (ab91021, Abcam, Cambridge, UK) from 0.025 mg/ml to 0.4 mg/ml, with subsequent regression analysis showing acceptable linearity. Serum samples (2 μl) or purified IgA (0.2 μg) were digested overnight with trypsin, diluted with a solvent containing 2% ACN and 0.1% FA, and ionized using a spray voltage of 2300 V and a source temperature of 150 °C. Analyzer parameters were optimized for each peptide/transition pair to ensure maximum selectivity. Peptide separation was achieved with a Eksigent 150 × 0.75 mm, 3 μm, 100Å column, using a 30-min gradient, at a flow rate of 300 nl/min, with solvent A (solvent A: water with 0.1% FA) and solvent B (solvent B: 98% ACN with 0.1% FA). An LC gradient, 2% B for 1 min, from 2% B to 35% B in 13 min, then to 80% B in 4 min, held for 2 min and from 80% B to 2% B in 1 min and held for 10 min. Both Q1 and Q3 resolution were the chosen “Unit” (± 0.7 Da). The acquired MRM.wiff files were analyzed using MultiQuant™ software (Version 2.1) and peak area was determined for each glycopeptide/peptide. The relative abundance of glycopeptides (area) was normalized according to the abundance of unique peptide (area).

##### Western Blotting

Individual serum samples were diluted at 1:10, and 1 μl of each sample was analyzed using anti-human IgA_2_ Fc antibody (ab99798, Abcam). One pooled serum sample (from random 10 individuals, 1 μl) was used as internal control (the last well of each gel). Protein was separated by 10% SDS-PAGE gels, transferred to PVDF membranes (Milipore, Billerica, MA), and blocked for 1 h in 5% (w/v) skimmed milk powder in TBS-T (50 mm Tris, pH 7.5, 150 mm NaCl, 0.1% Tween20, pH 7.4). Primary antibody was diluted at 1:1000 with 5% (w/v) skimmed milk powder in TBS-T and was incubated overnight at 4 °C. Subsequent washing with TBS-T and incubation with a horseradish peroxidase-labeled goat anti-mouse secondary antibody (Jackson Immunoresearch, PA, 1:10,000 dilution with TBS-T) were followed by ECL detection (Merck). The densitometry of the band was analyzed using Quantity One image processing software (Bio-Rad).

##### Data Analysis

Graphs were generated with GraphPad Prism 6.0 (GraphPad Software, Inc.). MRM and Western blotting results were analyzed using nonparametric Mann-Whitney U tests.

## RESULTS

### 

#### 

##### N-glycopeptide of Target 40-kDa Band in LC and HCC

**Fig. 1.**
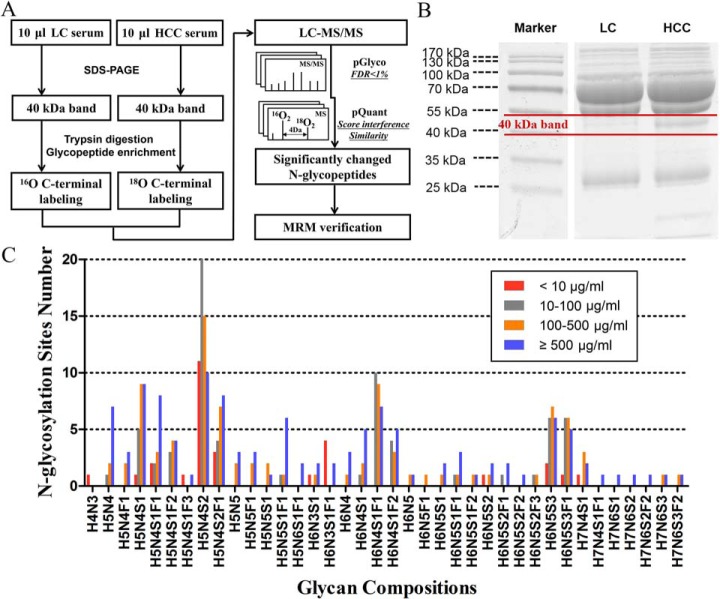
**N-glycopeptide of target 40 kDa-band detected in LC and HCC.** (*A*) The workflow in the study, pGlyco is designed for the identification and annotation of intact glycopeptides and improved pQuant can calculate the relative ^18^O/^16^O ratios. (*B*) Equal volumes of HCC (pooled from 10 individuals) and LC serum (pooled from 10 individuals) were acquired to separate 40 kDa-band. (*C*) Totally, 305 N-glycopeptides were detected and assigned to 38 kinds of N-glycan compositions. Comparison of the distribution of N-glycan compositions with attached sites in four glycoprotein concentration range. Glycoprotein concentration <10 μg/ml (red rectangle), 10–100 μg/ml (gray rectangle), 100–500 μg/ml (orange rectangle), and ≥500 μg/ml (blue rectangle).

We used an N-glycopeptide method based on ^18^O/^16^O C-terminal labeling to obtain comparisons of HBV-related HCC and LC. Fig. 1*A* shows the workflow in the study, and there is 4 Da mass shift between ^18^O- and ^16^O-labeled samples. pGlyco is designed for the identification and annotation of intact glycopeptides (21), and improved pQuant can calculate the relative ^18^O/^16^O ratios along with their interference scores (28). Considering the overlap of isotopic peaks of N-glycopeptides, a novel ratio algorithm has been embedded in pQuant (Supplemental Fig. S1). Standard haptoglobin was first used as model glycoprotein to evaluate the feasibility and stability of this method. A series of theoretical ratios of haptoglobin (10:1, 5:1, 2:1, 1:1, 1:2, 1:5, 1:10) was applied (three replicates) and dual-logarithm plots between theoretical ratios and experimental ratios showed a good linearity with correlation coefficient (R^2^) approximate of 0.99 (Supplemental Table S3).

Then, equal volume of HCC (pooled from 10 randomly selected HCC individuals) and LC serum (pooled from 10 randomly selected LC individuals) were acquired to separate 40-kDa band (Fig. 1*B*). In total, 305 N-glycopeptides were detected (Fig. 1*C*) and assigned to 38 kinds of glycan compositions. For example, H5N4S2 represented biantennary fully sialylated oligosaccharide, the most common composition in the detection. This composition occupied 56 N-glycosylation sites, and among them, 20 sites belonged to the serum glycoproteins with concentration 10–100 μg/ml. The detailed information of 305 N-glycopeptides including protein information (name, molecular weight, function, concentration), N-glycosylation site, and previously reported references are supplied in Supplemental Tables S4 and S5.

##### Two N-glycopeptides of IgA_2_ in LC and HCC

**Fig. 2.**
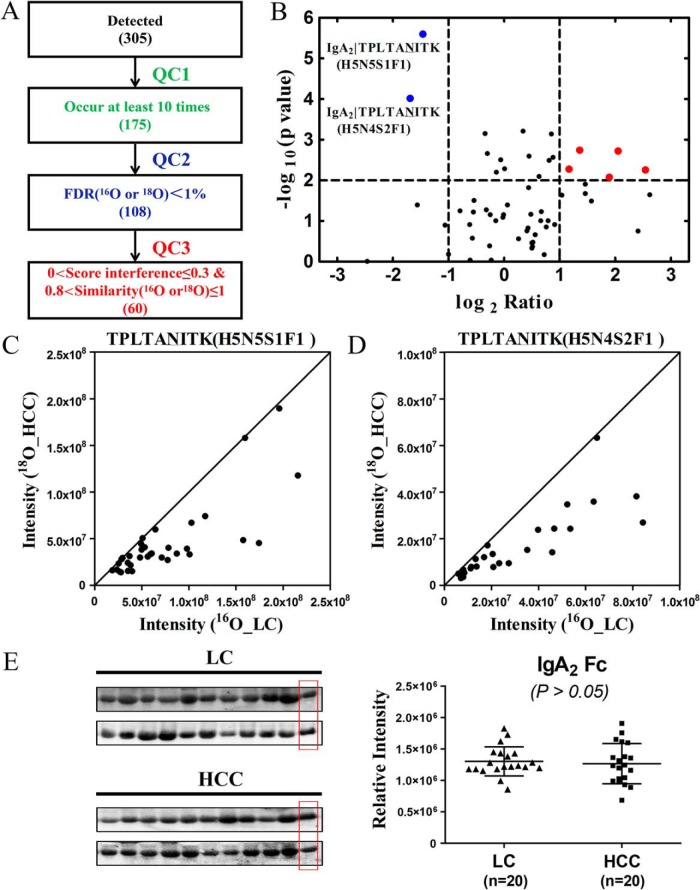
**TPLTAN^205^ITK (H5N5S1F1) and (H5N4S2F1) of IgA_2_ in LC and HCC.** (*A*) Based on 45 biological repeats, 60 N-glycopeptides that occurred at least 10 times (QC1) and passed through stringent filtering criteria (QC2, FDR<1%; QC3, 0<score interference ≤ 0. 3 and 0.8<similarity≤1) were considered. (*B*) TPLTAN^205^ITK (H5N5S1F1) and (H5N4S2F1) corresponding to the glycopeptides of IgA_2_ were decreased significantly in HCC compared with LC patients. (*C* and *D*) Another 37 biological repeats were performed to confirm the TPLTAN^205^ITK (H5N5S1F1) and (H5N4S2F1) alterations, respectively. (*E*) The protein level of IgA_2_ (20 individual LC and 20 individual HCC patients) was also evaluated using Western blotting, and the result showed there was no significant differences between LC and HCC. The last well of each gel was used as internal control.

Equal volumes of HCC (pooled from random 10 individuals) and LC serum (pooled from random 10 individuals) were acquired to separate the 40-kDa band, as one biological experiment. Based on 45 biological repeats (Fig. 2*A*), 60 N-glycopeptides that occurred at least 10 times (QC1) and passed stringent filtering criteria (QC2, FDR<1%; QC3, 0<score interference≤ 0.3 & 0.8 <similarity ≤1) were considered (Supplemental Tables S6). As shown in Fig. 2*B*, TPLTAN^205^ITK (H5N5S1F1) and (H5N4S2F1) corresponding to the glycopeptides of IgA_2_ (P01877) were decreased significantly in HCC compared with LC patients (*p* = 2.5E-06 and *p* = 9.5E-05, respectively). Only ^18^O-tagged of TPLTAN^205^ITK (H5N5S1F1) and (H5N4S2F1) showed there was no signal observed in the theoretical monoisotopic *m/z* of the ^16^O isoform, which guaranteed ^18^O incorporation efficiency (Supplemental Figs. S2 and S3).

TPLTAN^205^ITK (H5N5S1F1) may represent this N-glycosylation site attached with the monosialylated bisected fucosylated biantennary oligosaccharide and TPLTAN^205^ITK (H5N4S2F1) represents this site attached with the fucosylated biantennary fully sialylated oligosaccharide. Interestingly, increases in fucosylation and sialylation have been observed in patients with HCC (29, 30). Likewise, increased glycosylation such as carbohydrate antigen 19–9 was commonly elevated in the serum of patients with a variety of cancers, including pancreatic, gastric, and colorectal cancers (31).

**Fig. 3.**
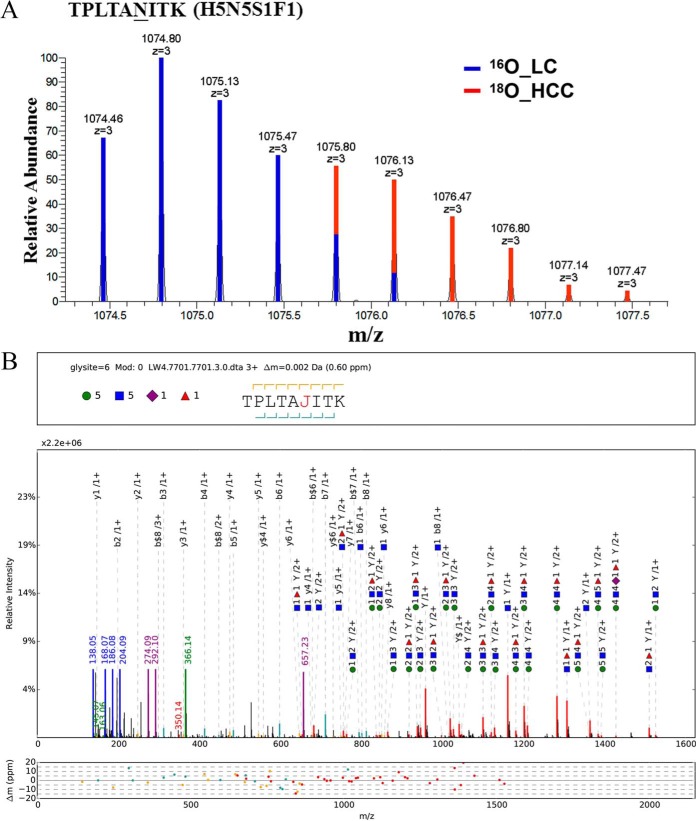
**Representative MS and pGlyco annotations of TPLTAN^205^ITK (H5N5S1F1).** (*A*) MS^1^ spectrum of TPLTAN^205^ITK (H5N5S1F1). pQuant reported that ^18^O/^16^O ratio was 0.4, both Similarity (^16^O) and Similarity (^18^O) were 0.96, and interference score was 0.03. (*B*) pGlyco annotations of TPLTAN^205^ITK (H5N5S1F1). MS^2^ spectrum was automatically annotated and displayed by the software tool gLabel embedded in pGlyco. “J” indicates the N-glycosylation site “N”; purple rhombus, sialic acid (S); green circle, hexose (H); blue square, N-acetylglucosamine (N); red triangle, fucose (F). The design of the upper box above each spectrum is glycan composition and peptide sequence. Peak annotation is shown in the middle box—green, blue, and purple peaks represent the fragment ions of the glycan moiety or the diagnostic glycan ions; red peaks represent the Y ions from glycan fragmentation; and yellow/cyan peaks represent the b/y ions from peptide backbone fragmentation. Mass deviations of the annotated peaks are shown in the lower box.

**Fig. 4.**
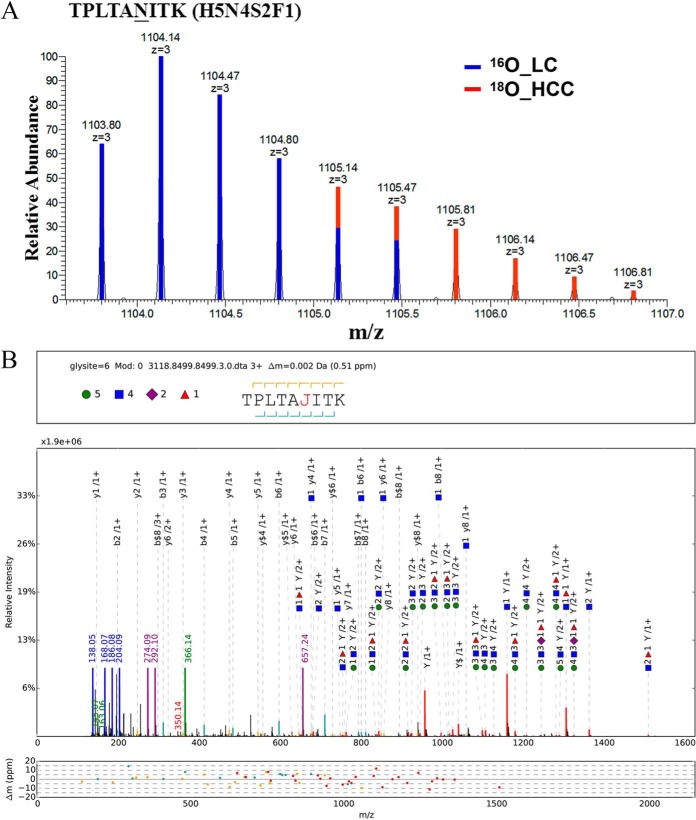
**Representative MS^1^ and pGlyco annotations of TPLTAN^205^ITK (H5N4S2F1).** (*A*) MS^1^ spectrum of TPLTAN^205^ITK (H5N4S2F1). pQuant reported that ^18^O/^16^O ratio was 0.3, the Similarity (^16^O) was 0.96, the Similarity (^18^O) was 0.95, and interference score was 0.03. (*B*) pGlyco annotations of TPLTAN^205^ITK (H5N4S2F1). MS^2^ spectrum was automatically annotated and displayed by the software tool gLabel embedded in pGlyco. The symbols were the same as those in Fig. 3*B*.

Another 37 biological repeats were performed to confirm the two N-glycopeptides alterations (Figs. 2*C* and 2*D*). To avoid bias in sample processing, cross-labeling (HCC sample was labeled with ^16^O and LC sample with ^18^O) were also performed (Supplemental Fig. S4). Results of purified IgA from pooled HCC and pooled LC patients (Supplemental Fig. S5, purification of IgA was confirmed by MALDI-TOF/TOF MS) also indicated TPLTAN^205^ITK (H5N5S1F1) and (H5N4S2F1) decreased considerably at N-glycopeptide level in HCC compared with LC patients. The protein level of IgA_2_ was also evaluated using Western blotting, and the result showed its protein concentration did not contribute to the variation in glycopeptide abundance (Fig. 2*E*). Representative MS spectra and pGlyco annotations of TPLTAN^205^ITK (H5N5S1F1) and (H5N4S2F1) are shown in Figs. 3 and 4.

##### MRM Validation of TPLTAN^205^ITK (H5N5S1F1) and (H5N4S2F1)

**Fig. 5.**
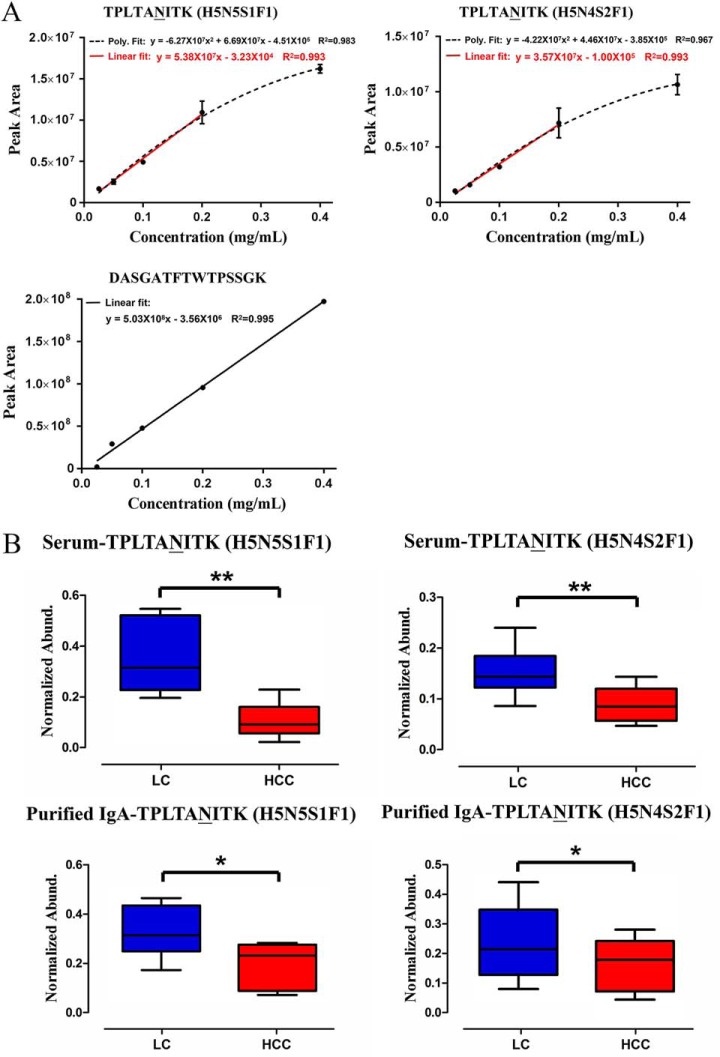
**MRM validation of TPLTAN^205^ITK (H5N5S1F1) and (H5N4S2F1) in LC and HCC.** (*A*) Target glycopeptides and unique peptide DASGATFTWTPSSGK of IgA_2_ were fitted linearly with R^2^ of 0. 99. (*B*) Glycopeptide abundance was divided by unique peptide abundance to separate out the contribution of protein concentration, named as Normalized Abund. Crude serum (10 HCC individuals and 10 LC individuals) and purified IgA (10 HCC individuals and 10 LC individuals) were applied to this approach, respectively. TPLTAN^205^ITK (H5N5S1F1) and (H5N4S2F1) were significantly decreased in HCC compared with LC patients. *p < 0.05, **p < 0.01.

MRM was used to monitor glycopeptide TPLTAN^205^ITK (H5N5S1F1) and (H5N4S2F1) normalized to absolute protein concentration in this study. Unique peptide DASGATFTWTPSSGK was chosen for IgA_2_ protein measurement and allowed unambiguous discrimination from any other IgA family member. MRM transitions used to monitor glycopeptides and peptides are provided in Supplemental Table S2, and the calibrations were fitted linearly with R^2^ of 0.99 (Fig. 5*A*).

**Fig. 6.**
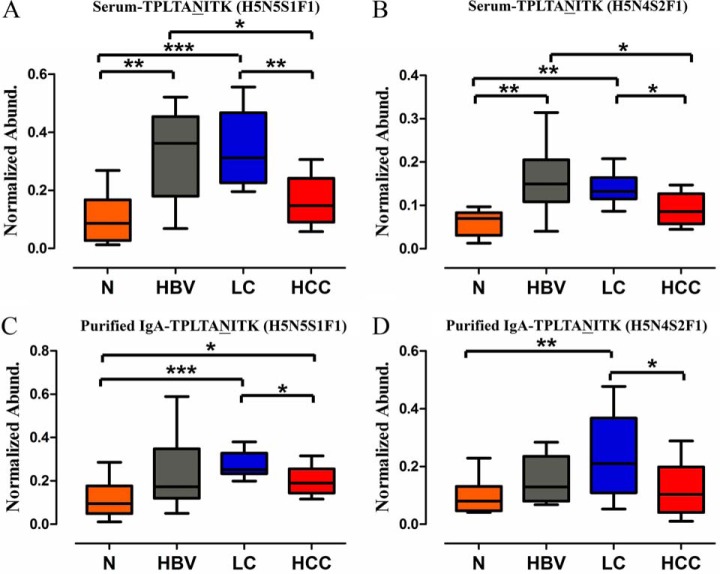
**TPLTAN^205^ITK (H5N5S1F1) and (H5N4S2F1) in normal control-HBV-LC-HCC cascade.** (*A* and *B*) Crude serum of 10 independent HCC, 10 independent LC patients, 10 HBV patients, and 10 normal subjects were enrolled using MRM and profound increases of the two glycopeptides were found from HBV infection. (*C* and *D*) Purified IgA of 10 independent HCC, 10 independent LC patients, 10 HBV patients, and 10 normal subjects were enrolled using MRM, and similar trend was found. *p < 0.05, **p < 0.01, ***p < 0.001.

Glycopeptide abundance was divided by unique peptide abundance to separate out the contribution of protein concentration (32). Crude serum (10 HCC individuals and 10 LC individuals) and purified IgA (10 HCC individuals and 10 LC individuals) were applied to this approach, respectively. Fig. 5*B* indicates that TPLTAN^205^ITK (H5N5S1F1) and (H5N4S2F1) were significantly decreased in HCC as compared with LC patients. Furthermore, crude serum and purified IgA of 10 independent HCC, 10 independent LC patients, 10 HBV patients, and 10 normal subjects were enrolled using MRM, and profound increases of the two glycopeptides were found in patients with HBV infection and LC patients (Fig. 6, Supplemental Tables S7). In addition, the variation in the two glycopeptides abundance was not caused by protein concentration. Thus, TPLTAN^205^ITK (H5N5S1F1) and (H5N4S2F1) might be part of a unique glycan signature indicating IgA-mediated mechanism and providing potential diagnostic clues in HBV-related liver diseases.

## DISCUSSION

The vast majority of the glycoproteins in serum are produced either by hepatocytes or by Ig-secreting plasma cells. Changes in the serum N-glycome profiles mainly reflect changes in liver or B-lymphocyte physiology. Callewaert *et al.* developed GlycoCirrhoTest (33) and GlycoFibroTest (34) from total N-glycome to specifically diagnose LC at an early stage. Most importantly, they detected an increase in the modification of serum fucosylated N-glycan with a bisecting GlcNAc residue in cirrhosis. Glycosylation of Igs plays a key role in the regulation of immune reactions such as binding affinities of antigens, receptors, and glycan-binding proteins (2). For IgA N-glycosylation, it was reported to bind pathogens and mediate clearance (35, 36). For example, N-glycans in IgA can play an important role in the clearance from blood and uptake by the liver (37). Serum IgA exists as two isotypes, IgA_1_ and IgA_2_. IgA nephropathy is histologically characterized by the deposition of IgA_1_ with undergalactosylation of O-glycans. However, these glycoforms in IgA_1_ are not restricted to IgA nephropathy (38). Prior research has suggested that Igs, including IgA, are the major glycoproteins involved in the modification of total serum N-glycome in LC (39, 40). Few studies have investigated detailed glycosylation changes of IgA_2_ (2, 41). Although there were many glycoforms identified for IgA_2_ Asn205 (Supplemental Table S5), only H5N5S1F1 and H5N4S2F1 on Asn205 passed the criteria and were quantified (Supplemental Table S6). Our study found the two N-glycopeptides alteration in HBV-related liver diseases, that is, dramatic increases in HBV infection and cirrhosis. In addition, the effects of the changes in glycosylation were not attenuated by the protein abundances. The results indicated that these increases were associated with HBV infection and cirrhosis and seemed to have no specific relationship with tumor. The emergence of antibody to HBsAg is usually related with protection against HBV or HBsAg clearance. Antibody glycosylation profiling at the site-specific level is expected to provide valuable new insights into the modulatory role of Ig glycosylation during immunological processes (2).

Glycosylation analyses based on MS commonly include glycosylation site, released glycan, and intact glycopeptide analyses (42). Compared with glycosylation site and released glycan analyses, profiles of intact glycopeptide enable correlation of glycan variants with specific site and pose a great challenge for method choice. For example, optimized collision-induced dissociation fragmentation enables data-independent acquisition of IgG intact glycopeptide, in which pooled plasma samples (*n* = 5) were used (43); product ion monitoring-based method was applied to glycopeptides quantification in the chromatographic peaks, in which individual anion exchange runs for different biological fluids (*n* = 6 and 7) were performed (44); direct peak areas of extracted ion chromatogram from each glycopeptide were obtained using 6 individual HCC samples (45) or samples pooled from 10 individuals (46); in addition, a novel analysis of variance-based mixed effects model for esophageal adenocarcinoma (*n* = 15), high-grade dysplasia (*n* = 12), and Barrett's disease (*n* = 7), as well as age and sex-matched disease-free (*n* = 15) subjects (47); Integrated GlycoProteome Analyzer was developed for label-free quantification of intact glycopeptides in pooled HCC (*n* = 10) and pooled normal (*n* = 10) samples (48). Recently, intact glycopeptides of innovator and biosimilar samples of Etanercept (non-IgG therapeutic protein) were quantified using ^18^O/^16^O labeling (49). However, ^18^O/^16^O labeling method was rarely used for quantification of intact glycopeptides in clinical samples.

The strength of labeling approach is its superior accuracy of quantification (50), and the label-free system is more compatible with sample detection in clinic (51). The combination of ^18^O/^16^O labeling method and MRM approach in this study can improve the reproducibility of the analytical platform for clinical samples. Through stringent filtering criteria, significantly increased TPLTAN^205^ITK (H5N5S1F1) and (H5N4S2F1) of IgA_2_ were detected in LC based on 82 comparisons for HCC and LC patients. In addition, N-glycopeptide alterations in normal-hepatitis-LC-HCC cascade were also investigated, and profound increases of the two N-glycopeptides were found in patients from HBV infection. For glycoform abundance evaluation on IgA_2_ Asn205, H5N5S1F1 was almost twofold in amount compared with H5N4S2F1 (Supplemental Table S6). Taken together, specific N-glycan alterations at IgA_2_ might be part of a unique glycan signature indicative of an IgA-mediated mechanism in liver diseases, and further analyses would be needed for any definitive conclusions.

## DATA AVAILABILITY

Partial mass spectrometric data and analyzed result datasets including the annotated spectra for all identified glycopeptides have been deposited in iProX (http://www.iprox.org) (52), which is an official member of ProteomeXchange Consortium. The project ID is IPX0001587000.

## Supplementary Material

Revised Supplemental Figures 8.8

Revised Supplementary Table 8.8

## Footnotes

^1^ The abbreviations used are:

HCC: hepatocellular carcinoma
FDR: false discover rate
HBV: hepatitis B virus
MRM: multiple reaction monitoring
ACN: acetonitrile
FA: formic acid.
