# Supplementary material for: N-glycopeptide Signatures of IgA2 in Serum from Patients with Hepatitis B Virus-related Liver Diseases
Source: Mol Cell Proteomics. 2019 Sep 9;18(11):2262–72. doi: 10.1074/mcp.RA119.001722 (PMC6823847; doi:10.1074/mcp.RA119.001722)
Supplement: Revised Supplemental Figures 8.8 [file 155086_0_supp_375466_pvwymr.pdf]

Supplementary Figures

$$Ratio1 = \frac{I_0}{I_2 + I_4 - \left(\frac{M_2}{M_0}\right) I_2 - \left[\left(\frac{M_2}{M_0}\right) + \left(\frac{M_4}{M_0}\right) - \left(\frac{M_2}{M_0}\right)^2\right] I_0}$$

$$Ratio2 = \frac{I_1}{I_3 + I_5 - \left(\frac{M_3}{M_1}\right) I_3 - [\left(\frac{M_3}{M_1}\right) + \left(\frac{M_5}{M_1}\right) - (\frac{M_3}{M_1})^2] I_1}$$

$$Ratio1' = \frac{I_1 \times \frac{M_0}{M_1}}{I_2 + I_4 - \left(\frac{M_2}{M_0}\right) I_2 - [\left(\frac{M_2}{M_0}\right) + \left(\frac{M_4}{M_0}\right) - (\frac{M_2}{M_0})^2] I_0}$$

$$Ratio2' = \frac{I_0 \times \frac{M_1}{M_0}}{I_3 + I_5 - \left(\frac{M_3}{M_1}\right) I_3 - [\left(\frac{M_3}{M_1}\right) + \left(\frac{M_5}{M_1}\right) - (\frac{M_3}{M_1})^2] I_1}$$

$$\text{ratio}\left(\frac{O_{16}}{O_{18}}\right) = \frac{ratio1 + ratio2 + ratio1' + ratio2'}{4}$$

Supplementary Figure S1. Parameter modification and novel ratio algorithm embedded in pQuant to quantify N-glycopeptides

For pQuant, considering the high molecular weight of N-glycopeptide, parameter EMASS_CUTOFF_PRO, which was the threshold of the isotopic peaks kept in the cluster, was set to 0.01. It meant only very small peaks (whose intensities were smaller than 1% of the whole cluster) were removed from quantification. pQuant used this parameter to control the number of isotopic peaks; considering the overlap of isotopic peaks of N-glycopeptides, novel ratio algorithm has been embedded in pQuant. It can provide effective correction and calculation for accuracy quantification of N-glycopeptides.

I_0, I_2, and I_4 are the measured relative intensities of the monoisotope peak for the peptide (Two ^{16}O labelling), the peak with 2 Da increase in mass (One ^{16}O labelling, one ^{18}O labelling), the peak with 4 Da increase (Two ^{18}O labelling) in mass, respectively.

M_0, M_2 and M_4 are the corresponding theoretical relative intensities of the isotopic envelope of the peptide, which are calculated using MS-Isotope (http://prospector.ucsf.edu).

I_1, I_3 and I_5 are the measured relative intensities of the isotope peak with 1 Da increase in mass of I_0, I_2, and I_4, respectively.

M_1, M_3 and M_5 are the corresponding theoretical relative intensities of the isotopic envelope of the peptide, which are calculated using MS-Isotope (http://prospector.ucsf.edu).

A

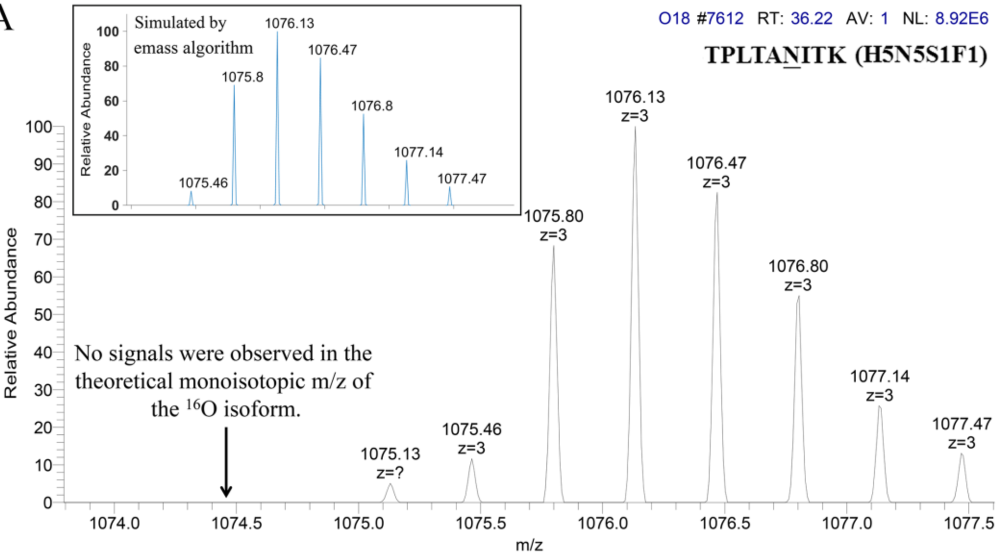

B

m/z	Measured Relative Abundance(%)	Simulated Relative Abundance(%)					
1075.13	5.1	7.1	6.3	5.6	4.8	4.1	0.0
1075.46	11.6	16.6	14.9	13.3	11.6	9.9	8.3
1075.80	68.2	73.9	73.0	72.0	71.1	70.1	69.2
1076.13	100.0	100.0	100.0	100.0	100.0	100.0	100.0
1076.47	83.0	82.8	83.2	83.6	84.0	84.4	84.8
1076.80	56.3	50.7	51.1	51.4	51.8	52.2	52.6
1077.14	26.4	24.7	24.9	25.2	25.4	25.7	26.0
1077.47	13.4	10.0	10.1	10.3	10.5	10.6	10.8
**Theoretical Labelling Efficiency**		**90%**	**91%**	**92%**	**93%**	**94%**	**95%**
**Pearson Correlation Coefficient**		**0.99412**	**0.99592**	**0.99730**	**0.99828**	**0.99887**	**0.99901**

Supplementary Figure S2. ^{18}O-tagged of TPLTAN^{205}ITK (H5N5S1F1)

$H_2{}^{18}O$-treated sample was subjected to LC-MS/MS analyses without combination with $H_2{}^{16}O$-treated sample. (A) Only ^{18}O-tagged of TPLTA**N**^{205}ITK (H5N5S1F1) showed there was no signal observed in the theoretical monoisotopic m/z of the ^{16}O isoform. Simulated MS by emass algorithm was also provided. (B) The labeling efficiency of this N-glycopeptides was simulated by emass algorithm, which showed

95% for TPLTA**N**^{205}ITK (H5N5S1F1).

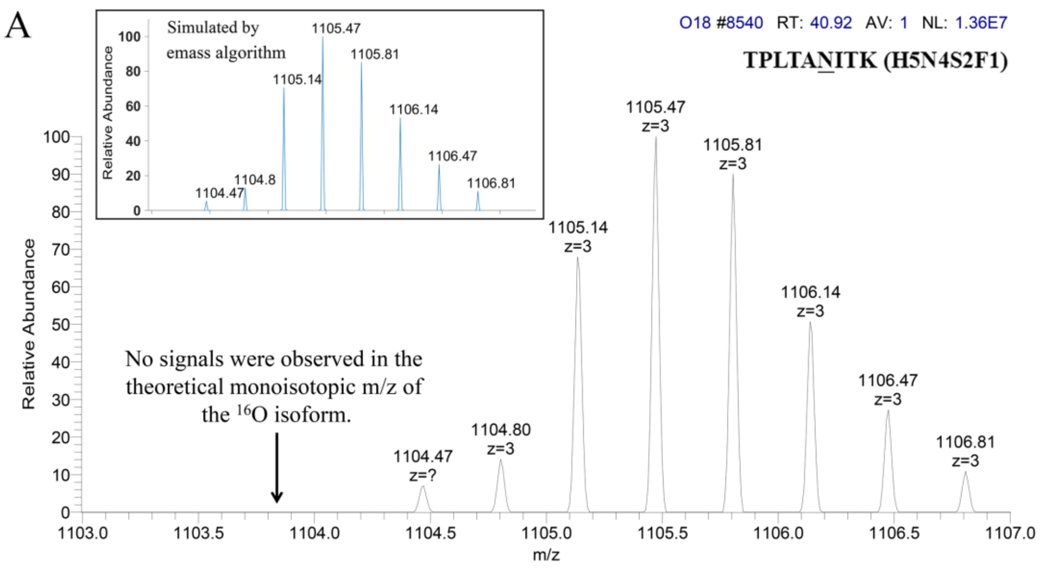

B

m/z	Measured Relative Abundance(%)	Simulated Relative Abundance(%)					
1104.47	7.2	6.9	6.2	5.4	4.7	4.0	0.0
1104.80	14.1	16.4	14.8	13.1	11.5	9.8	8.2
1105.14	69.0	72.6	71.7	70.7	69.7	68.8	67.8
1105.47	100.0	100.0	100.0	100.0	100.0	100.0	100.0
1105.81	89.3	84.4	84.8	85.2	85.7	86.1	86.5
1106.14	51.4	52.7	53.1	53.5	53.9	54.3	54.7
1106.47	27.4	26.1	26.4	26.7	27.0	27.3	27.6
1106.81	10.8	10.8	10.9	11.1	11.3	11.5	11.6
**Theoretical Labelling Efficiency**		**90%**	**91%**	**92%**	**93%**	**94%**	**95%**
**Pearson Correlation Coefficient**		**0.99762**	**0.99830**	**0.99857**	**0.99846**	**0.99798**	**0.99621**

Supplementary Figure S3. ^{18}O-tagged of TPLTAN**^{205}ITK (H5N4S2F1)**

$H_2{}^{18}O$-treated sample was subjected to LC-MS/MS analyses without combination with $H_2{}^{16}O$-treated sample. (A) Only ^{18}O-tagged of TPLTA**N**^{205}ITK (H5N4S2F1) showed there was no signal observed in the theoretical monoisotopic m/z of the ^{16}O isoform. Simulated MS by emass algorithm was also provided. (B) The labeling efficiency of this N-glycopeptides was simulated by emass algorithm, which showed 92% for TPLTA**N**^{205}ITK (H5N4S2F1).

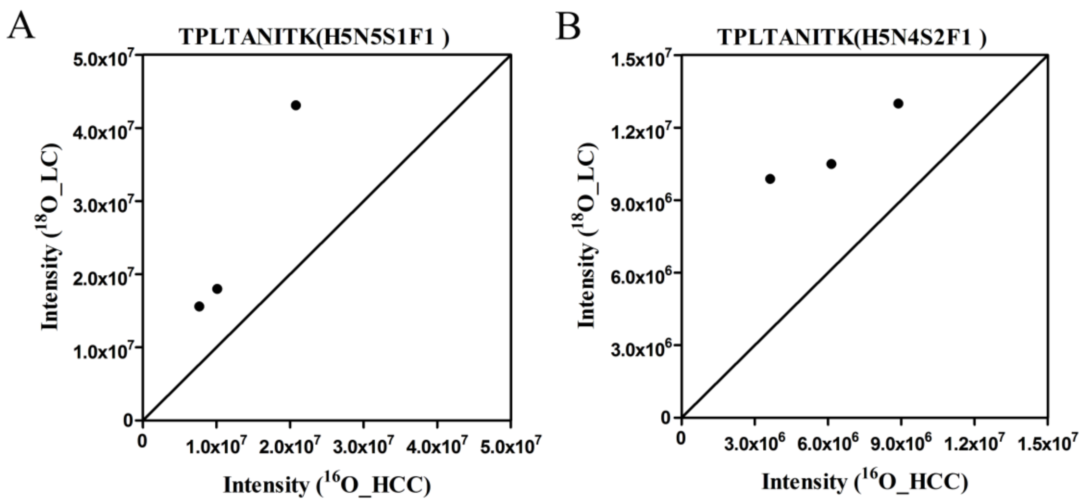

Supplementary Figure S4. Cross-labelling to investigate alterations of TPLTA__N__^{205}ITK (H5N5S1F1) and (H5N4S2F1)

To avoid bias in sample processing, cross-labelling (HCC sample was labelled with ^{16}O and LC sample with ^{18}O) were also supplied. Equal volume of HCC (pooled from 10 randomly selected HCC individuals) and LC serum (pooled from 10 randomly selected LC individuals) were acquired to separate 40 kDa-band, as one biological experiment. Totally, 3 biological repeats were performed. Both (A) TPLTA**N**^{205}ITK (H5N5S1F1) and (B) TPLTA**N**^{205}ITK (H5N4S2F1) increased significantly in HBV-related LC patients.

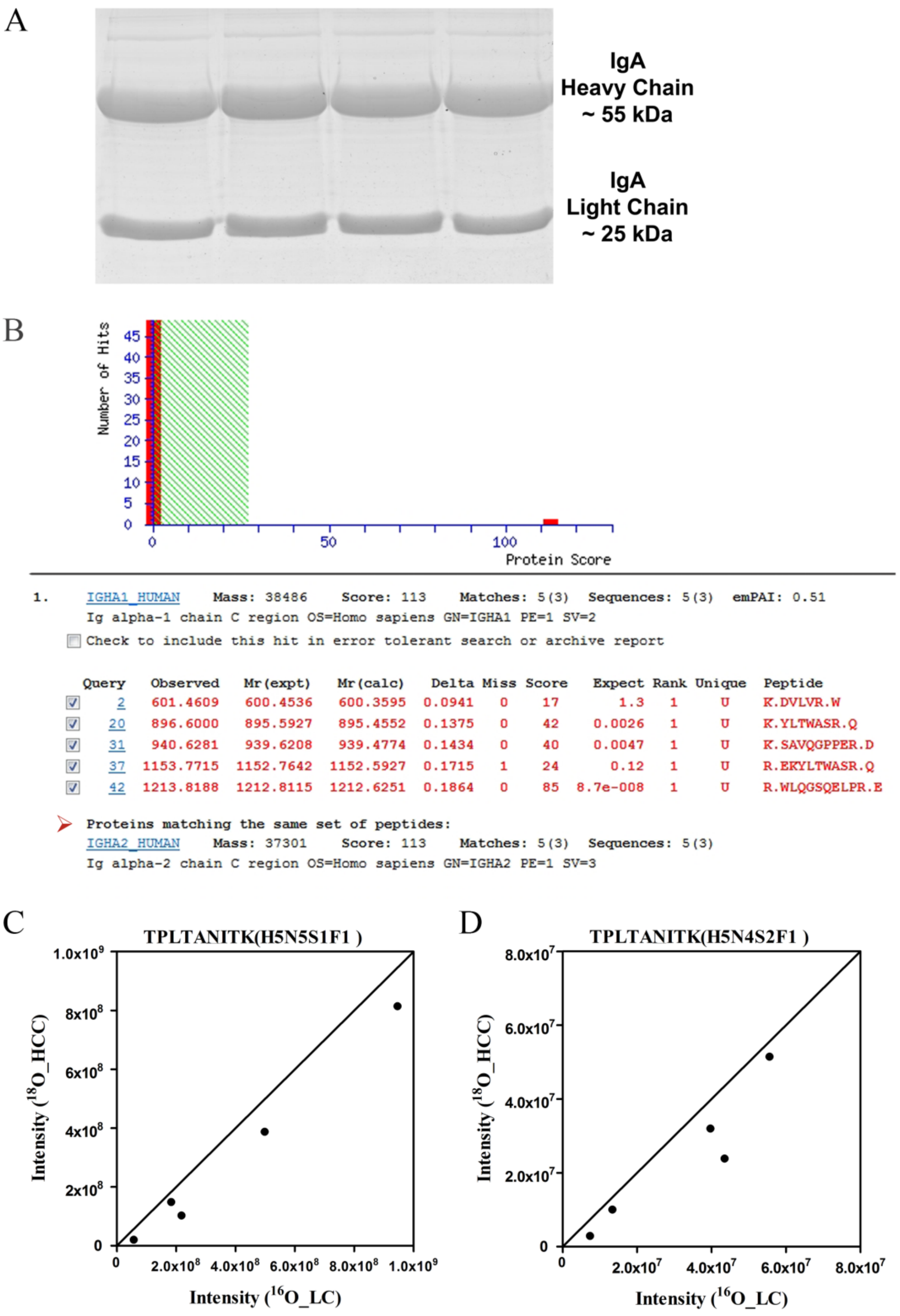

Supplementary Figure S5. Purification of IgA and quantification of the two glycopeptides from purified IgA based on $^{18}O/^{16}O$ labelling

(A) IgA was purified and separated by 10% SDS-PAGE. (B) The band of IgA was excised, reduced, alkylated and trypsin-treated. The tryptic peptides were extracted and applied to MALDI-TOF/TOF MS. The results confirmed these protein gels

corresponded to IgA. (C&D) Purified IgA from pooled HCC (^{18}O-tagged) and pooled LC patients (^{16}O-tagged) also indicated TPLTA**N**$^{\mathbf{205}}$ITK (H5N5S1F1) and (H5N4S2F1) decreased considerably at N-glycopeptide level in HCC compared with LC patients (5 biological repeats).
